# Association between admission albumin levels and 30-day readmission after hip fracture surgery in geriatric patients: a propensity score-matched study

**DOI:** 10.1186/s12891-024-07336-x

**Published:** 2024-03-25

**Authors:** Wanyun Tang, Wei Yao, Wei Wang, Wenbo Ding, Xiaomin Ni, RenJian He

**Affiliations:** 1 Department of Orthopedics, Zigong First People’s Hospital, Zigong, China; 2 Department of Orthopedics, Dandong Central Hospital, China Medical University, Dandong, China; 3 Department of Orthopedics, Zigong Fourth People’s Hospital, Zigong, China

**Keywords:** Hip fracture, Pneumonia, Hypoalbuminemia, Risk factor, Geriatric

## Abstract

**Purpose:**

This study aimed to evaluate the correlation admission albumin levels and 30-day readmission after hip fracture surgery in geriatric patients.

**Methods:**

In this retrospective cohort study, 1270 geriatric patients admitted for hip fractures to a level I trauma center were included. Patients were stratified by clinical thresholds and albumin level quartiles. The association between admission albumin levels and 30-day readmission risk was assessed using multivariate logistic regression and propensity score-matched analyses. The predictive accuracy of albumin levels for readmission was evaluated by ROC curves. The dose–response relationship between albumin levels and readmission risk was examined.

**Results:**

The incidence of 30-day readmission was significantly higher among hypoalbuminemia patients than those with normal albumin levels (OR = 2.090, 95%CI:1.296–3.370, *p* = 0.003). Furthermore, propensity score-matched analyses demonstrated that patients in the Q2(35.0–37.9 g/L) (OR 0.621, 95%CI 0.370–1.041, *p* = 0.070), Q3(38.0–40.9 g/L) (OR 0.378, 95%CI 0.199–0.717, *p* < 0.001) and Q4 (≥ 41 g/L) (OR 0.465, 95%CI 0.211–0.859, *p* = 0.047) quartiles had a significantly lower risk of 30-day readmission compared to those in the Q1(< 35 g/L) quartile. These associations remained significant after propensity score matching (PSM) and subgroup analyses. Dose–response relationships between albumin levels and 30-day readmission were observed.

**Conclusions:**

Lower admission albumin levels were independently associated with higher 30-day readmission rates in elderly hip fracture patients. Our findings indicate that serum albumin may assist perioperative risk assessment, and prompt correction of hypoalbuminemia and malnutrition could reduce short-term readmissions after hip fracture surgery in this high-risk population.

**Supplementary Information:**

The online version contains supplementary material available at 10.1186/s12891-024-07336-x.

## Introduction

Hip fractures in the elderly are a relatively common type of fracture, particularly among those aged 65 and older [1–3]. With the increasing aging of the population, the incidence of hip fractures is on the rise and over 4.5 million hip fracture patients worldwide are projected by 2050 [4]. This phenomenon has prompted the need for research on issues related to hip fractures in the elderly to better understand their epidemiological characteristics and risk factors. Hip fractures not only cause significant physical and psychological trauma but also come with a range of hazards and complications, including postoperative infections, pressure ulcers, deep vein thrombosis, and pulmonary embolism [5, 6]. Additionally, a patient's nutritional status can also impact their risk of postoperative complications after hip fracture surgery [7]. These complications not only prolong hospital stays but also increase the risk of readmission, making hip fractures a significant challenge in the healthcare system for the elderly [6, 8].

Hypoalbuminemia, defined as serum albumin < 35 g/L, is a common clinical condition typically characterized by serum albumin concentrations below the normal range [9]. It may indicate issues such as inflammation, protein loss, or malnutrition, and is associated with a range of adverse outcomes [10, 11]. Low albumin levels can result from various factors, including underlying medical conditions, liver disease, kidney problems, inflammation, malnutrition, and medication use [12, 13]. These factors may coexist in elderly hip fracture patients, affecting their albumin levels. Low albumin levels and malnutrition in hip fracture patients are associated with physiological and metabolic changes [14, 15].

Multiple previous studies have demonstrated an association between admission hypoalbuminemia and higher complication rates and mortality after hip fracture [16, 17]. For instance, a retrospective cohort study by Residori et al. found complications were associated with albumin level at post-surgery (no complications mean ± SD 26.2 ± 3.5 g/L; *n* = 80; complications mean ± SD 23.7 ± 3.6 g/L; *n* = 72; *p* < 0.001) [14]. Kieffer et al. in a cohort of 585 elderly hip fractures identified mortality was significantly higher in those with a low albumin level (odds ratio: 1.70, 95% confidence interval: 1.16–2.50, *p* = 0.0049) [16]. These findings underscore the need to further elucidate the relationship between perioperative hypoalbuminemia and outcomes in this population.

30-day readmission after hip fracture surgery is a common phenomenon (4–30%), especially among elderly patients [18–20]. The rate of 30-day readmission has significant implications for the utilization of healthcare resources and the process of patient recovery [21]. Therefore, understanding the epidemiological characteristics of readmission is crucial for improving medical management and preventing readmissions. Readmission after hip fracture is often accompanied by more severe complications, higher medical costs, and poorer recovery outcomes, placing a burden on both patients and the healthcare system [8, 22]. Hence, it is essential to identify factors that may influence readmission and implement appropriate interventions.

This study investigates the correlation between admission hypoalbuminemia and 30-day readmission incidence in patients with hip fractures. The findings from this study are anticipated to furnish healthcare practitioners with valuable insights into the management of hip fracture patients, specifically in terms of interventions targeting albumin levels and nutritional status. Moreover, this research aims to enhance our comprehension of the risk factors associated with readmission, with the ultimate goal of enhancing patient recovery and optimizing the utilization of healthcare resources.

## Methods

### Data sources and patient

This retrospective study using anonymized clinical data was approved by the Institutional Review Board (IRB) of the I trauma center. Following the study's observational design and use of deidentified data, the IRB waived the requirement for informed consent. Only anonymous data extracted from electronic medical records were analyzed, ensuring the protection of personal identifiable information. This retrospective analysis was conducted on patients who underwent hip fracture surgery between September 2011 and September 2023 at the level I trauma center, using continuous electronic health records.

Inclusion criteria were age ≥ 60 years, X-ray or CT diagnosis, and surgical confirmation. Exclusion criteria: (1) Absence of surgical intervention; (2) Age < 60 years; (3) Pathological, old, multiple or open fractures; (4) With severe infections or malignancies; (5) With severe cardiac/hepatic/renal dysfunction, and (6) Incomplete data.

### Data collection

Data were extracted from the hospital health information system. We collected a range of indicators in this study, including demographic, comorbidities, operation-related factors, laboratory findings, and common complications. Demographic data included gender, age, smoking status, and alcohol. Complications assessed were hypertension, diabetes, chronic obstructive pulmonary disease (COPD), cardiovascular disease, stroke, dementia, intracerebral hemorrhage, chronic liver disease, and chronic kidney disease. operation-related factors were hip fracture type, surgery type, intraoperative blood loss, transfusion, postoperative ICU, admission time, bedridden time, intraoperative time, and the American Society of Anesthesiologists (ASA) classification. In addition, laboratory findings included white blood cells, hemoglobin, red blood cell (RBC), white blood cells (RBC), Blood urea nitrogen (BUN), Creatinine (Cr), and albumin. Common complications included deep vein thrombosis (DVT), urinary tract infection (UTI), and pneumonia.

If a patient had multiple laboratory indicator measurements before surgery, we used the indicators closest to the admission time for analysis by default. Before starting the study, three researchers (WYT, YW, and RJH) received extensive training led by the principal investigator (RJH) focused on techniques for standardized data extraction from medical records, variable definitions, quality control procedures, and database entry. This intensive preparation emphasized best practices for reliable, consistent data collection. Subsequently, two researchers independently collected all variables, including 30-day readmission events, with any disagreements resolved through discussion and consensus or by the senior researcher (RJH).

### Exposure

The primary exposure was admission hypoalbuminemia, defined as a total protein level < 35 g/L. Blood samples were collected from hip fracture patients within 24 h of admission to determine the presence of baseline hypoalbuminemia. Normal albumin levels were defined as total albumin levels ≥ 35 g/L. To examine the dose–response relationship, albumin levels were categorized by clinical threshold into four groups normal albumin (C1 group): ≥ 35 g/L, mild hypoalbuminemia (C2 group): 34.9–30 g/L, moderate hypoalbuminemia (C3 group): 29.9–25 g/L, and severe hypoalbuminemia (C4 group): ≤ 24.9 g/L. Additionally, the albumin levels of the patients upon admission were divided into four groups based on quartiles: Q1 group (< 35 g/L), Q2 group (35.0–37.9 g/L), Q3 group (38.0–40.9 g/L), Q4 group (≥ 41 g/L).

### Outcome

The primary outcome of this study is 30-day readmission in patients with hip fractures. 30-day readmission was defined as any unplanned inpatient admission to an acute care hospital within 30 days of discharge from the index hip fracture surgery hospitalization. The 30-day readmission data were obtained through a review of electronic medical records. The date of discharge from the index hip fracture surgery was defined as day 0. The occurrence of readmission within 30 days of discharge was documented as a binary variable (yes/no).

### Statistical analysis

For descriptive statistics, categorical variables were summarized as percentages (%) and compared between groups using chi-square tests. Continuous variables were expressed as mean ± standard deviation (SD) and analyzed using independent sample t-tests between the two groups. To address missing data, we employed multiple imputation techniques because this approach provides less biased estimates than other methods for dealing with missing values [23].

To investigate the relationship between serum albumin levels and 30-day readmission, we used logistic regression analysis. In the univariate logistic regression analysis, we adjusted for potential confounding factors with p-values ≥ 0.05 and included variables with p-values < 0.05 in the subsequent multivariate logistic regression analysis. To evaluate the predictive accuracy of serum albumin levels for 30-day readmission, receiver operating characteristic (ROC) curves were constructed. The area under the ROC curve (AUC) was calculated to assess the capacity of serum albumin levels to distinguish between readmitted and non-readmitted patients.

To minimize the potential confounding effects of covariates, we used PSM with the nearest neighbor algorithm to match covariates in a 1:1 ratio between groups and used a caliper width of 0.25 standard deviations (SD) to compare group characteristics using standardized mean differences (SMDs). We matched on all 31 measured variables between the low albumin and normal albumin groups in this study. A subgroup analysis was conducted in the PSM cohort to further explore the diagnostic utility of albumin. The cohort was stratified by all covariates, and univariate logistic regression analysis was performed to determine the odds ratio (OR) and 95% confidence interval (CI) for the association between high albumin levels and 30-day readmission.

We also categorized albumin levels by clinical threshold into four groups (C1, C2, C3, and C4) and stratified patients into quartiles (Q1, Q2, Q3, and Q4) based on albumin levels to precisely assess the albumin-readmission dose–response relationship.

SPSS version 26(IBM Corp., Armonk, New York, USA) and R software version 4.0.3 (R Foundation for Statistical Computing, Vienna, Austria) are used to conduct all statistical analyses.

## Results

### Study population and baseline characteristics

**Fig. 1:**
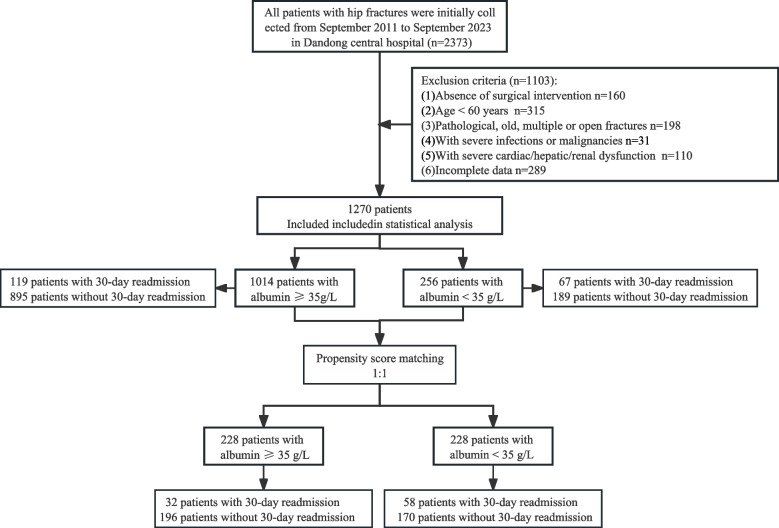
Flow diagram of enrollment

In this retrospective study, 1,270 patients were enrolled (Fig. 1). Table 1 summarizes the baseline characteristics of patients with and without 30-day readmission. Patients in the readmission group were of a higher mean age and had more comorbidities and complications. Supplementary eTable 1 shows the baseline characteristics of patients stratified into quartiles by admission albumin level.

**Table 1:** Baseline characteristics of the patients between 30-day readmission and non-readmission groups

Variables	Total patients (*n* = 1270)	Groups		^†^*P* for Trend
		Without readmission (*n* = 1084)	30-day readmission (*n* = 186)	
Demographic				
Male gender (n, %)	506 (39.8)	439(40.5)	67(36.0)	0.250
Age, × year (Mean, SD)	74.69(9.55)	73.79(9.46)	79.92(8.35)	< 0.001
Smoking (n, %)	218 (17.2)	191(17.6)	27(14.5)	0.300
Alcohol (n, %)	148(11.7)	130 (12.0)	18(9.7)	0.364
Comorbidities				
Hypertension (n, %)	636(50.1)	519(47.9)	117(62.9)	< 0.001
Diabetes (n, %)	291(22.9)	230(21.2)	61(32.8)	0.01
COPD (n, %)	149(11.7)	119(11.0)	30(16.1)	0.044
Cardiovascular disease (n, %)	390(30.7)	319(29.4)	71(38.2)	0.017
Stroke (n, %)	330(26.0)	252(23.2)	78(41.9)	< 0.001
Dementia(n, %)	48(3.8)	39(3.6)	9(4.8)	0.412
Intracerebral hemorrhage (n, %)	68(5.4)	51(4.7)	17(9.1)	0.013
Chronic liver disease (n, %)	58(4.6)	45(4.2)	13(7.0)	0.087
Chronic kidney disease (n, %)	64(5.0)	48(4.4)	16(8.6)	0.016
Operation				
Fracture type				
Femoral neck fracture (n, %)	679 (53.5)	575(53.0)	104(55.9)	0.560
Intertrochanteric fracture (n, %)	517(40.7)	443(40.9)	74(39.8)	
Subtrochanteric fracture (n, %)	74(5.8)	66(6.1)	8(4.3)	
Surgery type				
Total Hip Arthroplasty (n, %)	161(12.7)	139(12.8)	22(11.8)	< 0.001
Hemiarthroplasty (n, %)	319(25.1)	251(23.2)	68(36.6)	
Intramedullary nail fixation (n, %)	414(32.6)	355(32.7)	59(31.7)	
Internal fixation with steel plate (n, %)	168(13.2)	146(13.5)	22(11.8)	
Internal fixation with hollow nails (n, %)	208(16.4)	193(17.8)	15(8.1)	
Intraoperative blood loss, × ml (Mean, SD)	175.30(152.90)	173.97(153.55)	183.08(149.21)	0.453
Transfusion (n, %)	210(16.5)	175(16.1)	35(18.8)	0.365
Postoperative ICU (n, %)	63(5.0)	53(4.9)	10(5.4)	0.777
Admission time				
< 6 h (n, %)	672(52.9)	577(53.2)	95(51.1)	0.087
6–24 h (n, %)	195(15.4)	174(16.1)	21(11.3)	
≥ 24 h (n, %)	403(31.7)	333(30.7)	70(37.6)	
Bedridden time, × day (Mean, SD)	5.90(4.03)	5.85(4.07)	6.18(3.80)	0.299
Intraoperative time, × hour (Mean, SD)	1.67(0.80)	1.68(0.82)	1.58(0.68)	0.096
ASA classification				
III-IV (n, %)	710(55.9)	566(52.2)	144(77.4)	< 0.001
I-II (n, %)	560(44.1)	518(47.8)	42(22.6)	
Laboratory findings				
WBC count, × 10^9/L (Mean, SD)	8.84(2.86)	8.78(2.85)	9.16(2.88)	0.098
HGB level, × g/L (Mean, SD)	119.80(20.63)	120.77(20.44)	114.14(20.84)	0.102
Blood glucose, × mmol/L (Mean, SD)	6.92(2.65)	6.78(2.53)	7.79(3.12)	< 0.001
BUN, × mmol/L (Mean, SD)	7.39(4.85)	7.27(4.77)	8.14(5.22)	0.024
Cr, × umol/L (Mean, SD)	72.35(64.38)	70.99(58.47)	80.29(91.34)	0.069
D-Dimer, × mg/L (Mean, SD)	4.89(5.04)	4.82(5.01)	5.28(5.22)	0.241
Common complication				
DVT (n, %)	164(12.9)	130(12.0)	34(18.3)	0.018
UTI (n, %)	294(23.1)	238(22.0)	56(30.1)	0.015
Pneumonia (n, %)	115(9.1)	79(7.3)	36(19.4)	< 0.001

*Abbreviations: SD* Standard deviation, *COPD* Chronic obstructive pulmonary disease, *ASA* the American Society of Anesthesiologists Physical Status Classification System, *WBC* White blood cell, *HGB* hemoglobin, *RBC* red blood cell, *WBC* White blood cell, *BUN* Blood urea nitrogen, *Cr* Creatinine, *DVT* Deep Vein Thrombosis, *UTI* Urinary Tract Infection

^†^*P* values are from Fisher's exact test for continuous variables and from the chi-square test for categorical variables

### Propensity score matching

**Fig. 2:**
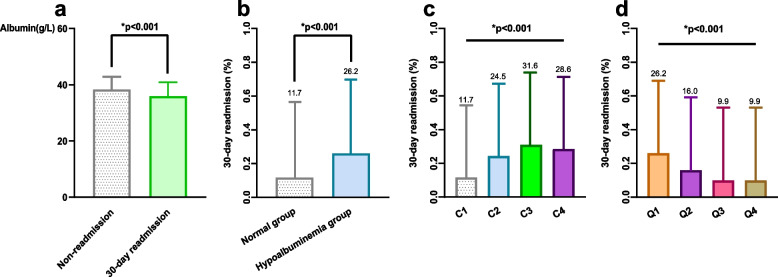
Relationship between different albumin level groups and 30-day readmission rates in patients with hip fracture. **a** Mean and standard deviation of albumin levels between the 30-day readmission group and non-readmission group. **b** Comparison of 30-day readmission rates between normal albumin group and hypoalbuminemia group. **c** Patients were categorized into 4 groups using clinical thresholds, comparing 30-day readmission rates among the 4 groups. **d** Patients were divided into 4 quartiles, comparing 30-day readmission rates among the 4 quartiles

The baseline characteristics of patients stratified into hypoalbuminemic and normoalbuminemic groups before and after 1:1 PSM are presented in Table 2. After PSM, standardized mean differences (SMDs) for all covariates decreased to below 0.1, indicating successful group matching. Before PSM, the 30-day readmission rate significantly differed between the normoalbuminemic group and the hypoalbuminemic group (11.7% vs. 26.2%, *p* < 0.001) (Fig. 2b and Table 3). Even after PSM, these results remained robust (14.0% vs. 25.4%, *p* = 0.002) (Table 3).

**Table 2:** Patient characteristics before and after propensity score matching by admission albumin levels (low < 35 g/L vs. normal ≥ 35 g/L)

Variables	Before PSM			After PSM		
	Albumin < 35 (*n* = 256)	Albumin ≥ 35 (*n* = 1014)	SMD	Albumin < 35 (*n* = 228)	Albumin ≥ 35 (*n* = 228)	SMD
Demographic						
Male gender (n, %)	111(43.4)	395(39.0)	0.089	95(41.7)	90(39.5)	0.045
Age, × year (Mean, SD)	78.58(9.53)	75.00(13.00)	0.655	74.00(17.00)	74.50(14.00)	0.019
Smoking (n, %)	31(12.1)	187(18.4)	0.177	29(12.7)	27(11.8)	0.027
Alcohol (n, %)	22(8.6)	126(12.4)	0.125	21(9.2)	23(10.1)	0.030
Comorbidities						
Hypertension (n, %)	138(53.9)	498(49.10)	0.096	125(54.8)	134(58.8)	0.080
Diabetes (n, %)	50(19.5)	241(23.8)	0.103	47(20.6)	53(23.2)	0.063
COPD (n, %)	39(15.2)	110(10.8)	0.130	33(14.5)	27(11.8)	0.078
Cardiovascular disease (n, %)	94(36.7)	296(29.2)	0.160	80(35.1)	76(33.3)	0.037
Stroke (n, %)	91(35.5)	239(23.6)	0.264	74(32.5)	73(32.0)	0.009
Dementia (n, %)	13(5.1)	35(3.5)	0.080	10(4.4)	7(3.1)	0.069
Intracerebral hemorrhage (n, %	13(5.1)	55(5.4)	0.015	13(5.7)	8(3.5)	0.105
Chronic liver disease (n, %)	17(6.6)	41(4.0)	0.116	15(6.6)	14(6.1)	0.018
Chronic kidney disease (n, %)	16(6.3)	48(4.7)	0.067	15(6.6)	20(8.8)	0.082
Operation						
Fracture type						
Femoral neck fracture (n, %)	91(35.5)	588(58.0)	0.410	86(37.7)	85(37.3)	0.036
Intertrochanteric fracture (n, %	146(57.0)	371(36.6)		126(55.3)	123(53.9)	
Subtrochanteric fracture (n, %)	19(7.4)	55(5.4)		16(7.0)	20(8.8)	
Surgery type						
Total Hip Arthroplasty (n, %)	24(9.4)	137(13.5)	0.011	22(9.6)	13(5.7)	0.026
Hemiarthroplasty (n, %)	52(20.3)	267(26.3)		48(21.1)	59(25.9)	
Intramedullary nail fixation (n, %)	113(44.1)	301(29.7)		96(42.1)	96(42.1)	
Internal fixation with steel plate (n, %)	48(18.8)	120(11.8)		44(19.3)	41(18.0)	
Internal fixation with hollow nails (n, %)	19(7.4)	189(18.6)		18(7.9)	19(8.3)	
Intraoperative blood loss, × ml (Mean, SD)	196.56(158.95)	169.94(150.94)	0.172	200.00(200.00)	196.00(200.00)	0.072
Transfusion (n, %)	64(25.0)	146(14.4)	0.269	55(24.1)	61(26.8)	0.060
Postoperative ICU (n, %)	26(10.2)	37(3.6)	0.258	19(8.3)	22(9.6)	0.046
Admission time						
< 6 h (n, %)	115(44.9)	557(54.6)	0.260	93(40.8)	84(36.8)	0.029
6–24 h (n, %)	32(12.5)	163(16.1)		30(13.2)	42(18.4)	
≥ 24 h (n, %)	109(42.6)	294(29.0)		105(46.1)	102(44.7)	
Bedridden time, × day (Mean, SD)	6.77(5.04)	5.68(3.70)	0.247	(3.00)	5.00(3.25)	0.019
Intraoperative time, × hour (Mean, SD)	1.77(0.86)	1.64(0.79)	0.153	1.53(1.08)	1.50(1.08)	0.031
ASA classification						
III-IV (n, %)	180(70.3)	530(52.3)	0.377	157 (68.9)	154(67.5)	0.028
I-II (n, %)	76(29.7)	484(47.7)		71(31.1)	74(32.5)	
Laboratory findings						
WBC count, × 10^9/L (Mean, S)	8.49(3.24)	8.85(2.75)	0.019			0.017
HGB level, × g/L (Mean, SD)	106.38(20.91)	123.19(19.13)	0.839	125.00(25.00)	122.50(17.75)	0.031
Blood glucose, × mmol/L (Mean, SD)	6.81(2.36)	6.95(2.71)	0.058	38.00(5.00)	38.00(6.00)	0.087
BUN, × mmol/L (Mean, SD)	8.12(4.15)	7.21(4.99)	0.198			0.008
Cr, × umol/L (Mean, SD)	74.23(48.61)	71.88(67.80)	0.040	125.00(25.00)	122.50(17.75)	0.033
D-Dimer, × mg/L (Mean, SD)	4.85(4.60)	4.89(5.14)	0.009	2.98(4.43)	4.24(7.45)	0.056
Common complication						
DVT (n, %)	41(16.0)	123(12.1)	0.112	35(15.4)	10(17.5)	0.059
UTI (n, %)	88(34.4)	206(20.3)	0.319	73(32.0)	78(34.2)	0.047
Pneumonia (n, %)	36(14.1)	79(7.8)	0.202	28(12.3)	29(12.7)	0.013

*Abbreviations: SMD* standardized mean difference, *SD* Standard deviation, *PSM* propensity score matching, *SD* Standard deviation, *COPD* Chronic obstructive pulmonary disease, *ASA* the American Society of Anesthesiologists Physical Status Classification System, *WBC* White blood cell, *HGB hemoglobin*, *RBC* red blood cell, *WBC* White blood cell, *BUN* Blood urea nitrogen, *Cr* Creatinine, *DVT* Deep Vein Thrombosis, *UTI* Urinary Tract Infection

**Table 3:** Comparison of the incidence of 30-day readmission before and after PSM based on admission albumin levels

Hematologic parameters	Clinical cutoffs	Before PSM		*p-value	Clinical cutoffs	After PSM		*p-value
		Without 30-day readmission	30-day readmission			Without 30-day readmission	30-day readmission	
Cutoff value	≥ 35 (*n* = 1014)	895(88.3)	119(11.7)	< 0.001	≥ 35 (*n* = 228)	196(86.0)	32(14.0)	0.002
	< 35 (*n* = 256)	189(73.8)	67(26.2)		< 35 (*n* = 228)	170(74.6)	58(25.4)	

*Abbreviations:* *PSM* propensity scores matching

^*^p-value is from Chi-Squared Test to indicate significant differentiation (*p* < 0.05 means significant differentiation)

### Association between serum albumin levels and 30-day readmission

Patients in the 30-day readmission group had significantly lower serum albumin levels at admission than those in the non-readmission group (Fig. 2a). A multivariate logistic regression model was employed to investigate the relationship between serum albumin levels at admission and 30-day readmission (Supplementary eTable 2). After adjusting for potential confounders, seven independent predictors of 30-day readmission were identified: age, stroke, intracerebral hemorrhage, ASA classification, blood glucose levels, serum albumin levels, and pneumonia.

Patients with hypoalbuminemia (serum albumin < 35 g/L) on admission had a significantly higher risk of 30-day readmission compared to those with normal albumin levels (≥ 35 g/L) (OR 2.666, 95% CI 1.902–3.738, *p* < 0.001). This association remained significant after adjusting for potential confounders in a multivariate logistic regression model (Table 4), with patients admitted with hypoalbuminemia having a nearly twofold increased odds of 30-day readmission (adjusted OR 1.974, 95% CI 1.340–2.910, *p* = 0.001). The results were consistent even after PSM to minimize selection bias (PSM-adjusted OR 2.090, 95% CI 1.296–3.370, *p* = 0.003). Furthermore, patients in group C2 (serum albumin 34.9–30 g/L) had a significantly higher incidence of 30-day readmission than those in group C1 (≥ 35 g/L) after PSM (*p* = 0.018).

**Table 4:** Unadjusted and adjusted association between admission albumin levels and 30-day readmission

Type	Albumin level (g/L)	Events, n (%)	Unadjusted OR	p* trend 1	Multivariable Regression adjusted OR	p* trend 2	PSM adjusted OR	p* trend 3
Continuous	Per 1	NA	1.112(1.075–1.149)	< 0.001	1.064(1.020–1.111)	0.003	NA	NA
Cutoff value	≥ 35	119(11.7)	1 [Reference]	< 0.001	1 [Reference]	0.001	1 [Reference]	0.003
	< 35	67(26.2)	2.666(1.902–3.738)		1.974(1.340–2.910)		2.090(1.296–3.370)	
Clinical threshold	C1(≥ 35)	119(11.7)	1 [Reference]	NA	1 [Reference]	NA	1 [Reference]	NA
	C2(34.9–30)	47(24.5)	2.438(1.666–3.567)	< 0.001	2.015(1.311–3.099)	0.001	1.908(1.117–3.259)	0.018
	C3(29.9–25)	18(31.6)	3.471(1.924–6.264	< 0.001	2.198(1.106–4.369)	0.004	1.791(0.687–4.671)	0.233
	C4(≤ 24.9)	2(28.6)	0.332(0.064–1.732)	0.191	0.586(0.099–3.474)	0.556	NA^#^	NA^#^
Quartile	Q1(< 35)	67(26.2)	1 [Reference]	NA	1 [Reference]	NA	1 [Reference]	NA
	Q2(35.0–37.9)	48(16.0)	0.538(0.355–0.803)	0.003	0.599(0.378–0.951)	0.029	0.621(0.370–1.041)	0.070
	Q3(38.0–40.9)	34(9.9)	0.311(0.198–0.489)	< 0.001	0.380(0.225, 0.641)	0.004	0.378(0.199–0.717)	< 0.001
	Q4(≥ 41)	37(9.9)	0.312(0.201–0.483)	< 0.001	0.515(0.293–0.908)	0.022	0.465(0.211–0.859)	0.047

The factors of the multivariable regression: Age, Stroke, Intracerebral hemorrhage, ASA classification, Blood glucose, Albumin, Pneumonia

*CI* confidence interval, *OR* odds ratio, *PSM* propensity scores matching

^*^P for trend; NA^#^, The number of individuals in the C4 group is too small to conduct propensity score matching

**Fig. 3:**
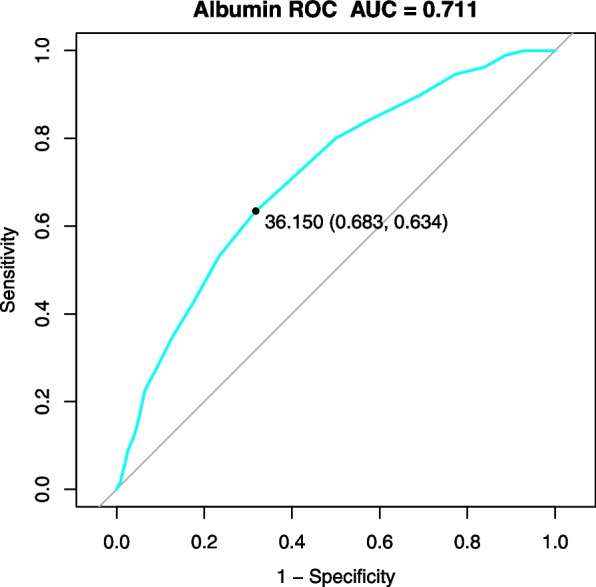
The prediction model of admission albumin levels for 30-day readmission rate after hip fracture

After adjusting for confounders in a multivariate regression model, patients in the higher serum albumin quartiles (Q2-Q4) had a significantly decreased risk of 30-day readmission compared to those in the lowest quartile (Q1) (Q2: adjusted OR 0.599, 95% CI 0.378–0.951, *p* = 0.029; Q3: adjusted OR 0.380, 95% CI 0.225–0.641, *p* = 0.004; Q4: adjusted OR 0.515, 95% CI 0.293–0.908, *p* = 0.022). This inverse association persisted after PSM to balance baseline characteristics across groups (Q2: PSM-adjusted OR 0.621, 95% CI 0.370–1.041, *p* = 0.070; Q3: PSM-adjusted OR 0.378, 95% CI 0.199–0.717, *p* < 0.001; Q4: PSM-adjusted OR 0.465, 95% CI 0.211–0.859, *p* = 0.047). Moreover, an AUC of 0.711 for the albumin ROC curve (Fig. 3), indicates the good predictive value of albumin levels as an independent biochemical marker to assess the risk of 30-day readmission in elderly hip fracture patients after discharge. Taken together, these findings indicate that higher serum albumin levels on admission are associated with a lower risk for 30-day readmission in this patient cohort.

### Dose–response relationship

**Fig. 4:**
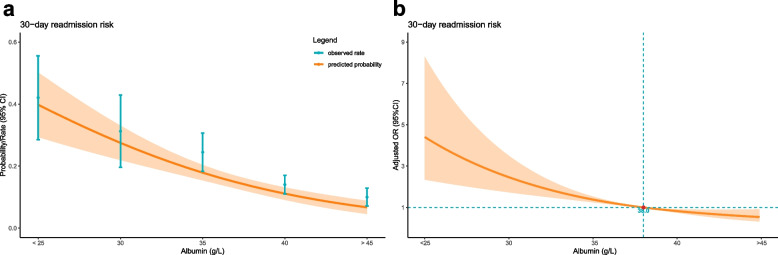
Relationship between admission albumin level and 30-day readmission in patients with hip fracture. **a** Predicted probabilities and the observed rate of 30-day readmission. **b** Adjusted odds ratios (ORs) and 95% confidence intervals (CIs) are shown for each 5 g/L deviation away from the reference value (35 g/L)

A dose-dependent relationship between admission hypoalbuminemia and 30-day readmission was observed, with a higher incidence of 30-day readmission associated with decreasing admission albumin levels (Fig. 2c, d**)**. The 30-day readmission rate exhibited a gradual upward trend as admission albumin levels declined (*p* < 0.001). Predicted probabilities and actual observed values for 30-day readmission based on admission albumin levels (Fig. 4a) demonstrate an increasing risk of 30-day readmission with lower baseline albumin. Compared to patients with admission albumin ≥ 38 g/L, those with levels < 38 g/L showed a higher 30-day readmission risk. Even when analyzed as a continuous variable, higher admission albumin levels remained associated with reduced 30-day readmission (Fig. 4b). Together, these data demonstrate admission hypoalbuminemia as an independent risk factor for 30-day readmission in a dose-dependent manner.

### Interaction analysis

**Fig. 5:**
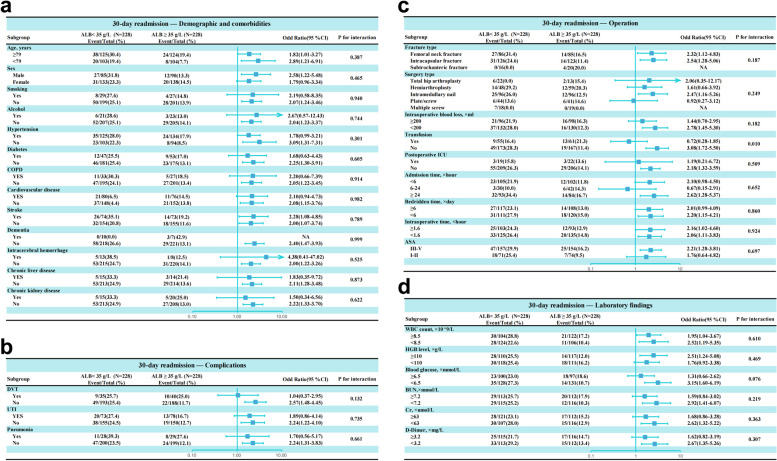
Subgroup analysis of association admission hypoalbuminemia and 30-day readmission after PSM. **a** Subgroup analysis of variables related to demographics and comorbidities; **b** Subgroup analysis of variables related to complications; **c** Subgroup analysis of variables related to operation. **d** Subgroup analysis of variables related to laboratory findings

To assess potential interactions between admission hypoalbuminemia and other variables, additional analyses were conducted (Fig. 5a, b, c, d). A significant interaction was observed between admission hypoalbuminemia and transfusion (interaction *P* = 0.010), suggesting the effect of hypoalbuminemia on 30-day readmission risk may differ based on transfusion status. However, no significant effect modification of the association between admission hypoalbuminemia and 30-day readmission was found for the other variables examined. These data indicate transfusion as a potential effector of the relationship between low admission albumin and increased 30-day readmission risk.

## Discussion

This retrospective study demonstrates that admission hypoalbuminemia is an independent risk factor for 30-day hospital readmission in elderly patients with hip fractures. The findings showed a significant association between lower serum albumin levels at admission and increased odds of 30-day readmission, even after adjusting for potential confounders. Furthermore, a dose–response relationship was observed, with decreasing admission albumin levels associated with a higher incidence of 30-day readmission.

The 30-day readmission rate of 14.6% found in this elderly hip fracture cohort aligns with readmission rates reported in other studies, which range from 4–30% [18–20]. In a retrospective cohort study of 5637 patients after geriatric hip fractures by Matt et al. found hypoalbuminemia was independently associated with a significant increase in readmission risk (11.4% vs. 4.1%; RR 2.53) [24]. In other large retrospective cohort study of 1856 patients with hip fracture patients, Borge et al. found Hypoalbuminemia is associated with 30-day mortality (OR 1.09, 95% CI 1.05;1.11 (*p* < 0.0001) [15]. Ryan et al. found 20,278 patients undergoing surgery for hip fracture, admission hypoalbuminemia (< 3.5 g/dL) was a powerful predictor of postoperative complications (*p* = 0.028) [17]. Jacob et al. conducted a study of 6138 patients and found that hypoalbuminemia was independently associated with the occurrence of readmission (RR, 1.54; 95% CI, 1.13–2.08; *P* = 0.006) [25]. Tian et al. analyzed 930 patients with hip fracture and discovered that low preoperative albumin levels were predictors of readmission (*p* < 0.05) in those patients [20]. Sim et al. investigated 971 patients after hip fracture surgery and found in comparison to the group with normal albumin concentration, low albumin (≤ 35 g/L) is prevalent in elderly hip fracture patients and is associated with slower recovery of function and quality of life [26]. Readmission after hip fracture surgery is a common occurrence, particularly in frail, elderly patients with multiple comorbidities. Our study confirms admission hypoalbuminemia as a predictor of 30-day readmission risk, likely related to poor nutritional status and physiological resilience.

Although the specific mechanism by which admission hypoalbuminemia causes 30-day readmissions in patients is not completely clear, previous research has suggested hypoalbuminemia indicates malnutrition and systemic inflammation, which can adversely impact wound healing, muscle strength, and immunity [13, 27]. Low albumin levels reduce colloid osmotic pressure, increase capillary permeability, and diminish antioxidant effects, thereby impairing postoperative recovery [28, 29]. Hypoalbuminemia also reflects underlying chronic disease, compounding the surgical risk [30–32]. Moreover, albumin synthesis may decline with aging due to reduced liver function [33, 34]. All these factors likely contribute to the higher readmission risk seen with lower admission albumin levels in our elderly hip fracture cohort.

Several interventions to correct hypoalbuminemia and malnutrition preoperatively have shown promise for reducing postoperative complications and readmissions. A randomized trial by Botella-Carretero et al. found that specialized preoperative nutritional support increased serum albumin levels and lowered complications in hip fracture patients [35]. Another study by Lee et al. implemented an interdisciplinary nutritional program starting from hospital admission, significantly improving albumin levels and reducing complication rates [36]. These findings indicate that timely nutritional optimization and albumin repletion perioperatively may help attenuate readmission risk. Further research is warranted to determine the optimal methods and duration of preoperative nutritional interventions.

Evidence suggests that preoperative nutritional intervention, when feasible before hip fracture surgery, can increase serum albumin levels and potentially lower post-operative complication risk [35, 36]. However, opportunities for such aggressive correction are often limited given guidelines recommending surgical fixation within 24–48 h [5]. Implementing comprehensive nutrition programs in this timeline can encounter barriers like resource constraints, staffing availability, and clinical inertia. Some research indicates that even nutrition initiation in the acute post-operative window may offer benefits following expedited surgery [37]. Ultimately, balancing efficacy with real-world feasibility will be key for solutions to leverage nutrition support in this population.

Subgroup analysis indicated transfusion as a potential effect modifier in the relationship between hypoalbuminemia and readmission. Transfusion may reflect greater injury severity or perioperative blood loss, amplifying the detrimental effects of low albumin. Several mechanisms may explain how transfusion could modify the effect of hypoalbuminemia on readmission risk. Firstly, transfusion may replenish plasma albumin levels, thereby correcting the low oncotic pressure and complications like edema and ascites that are associated with hypoalbuminemia [13]. Furthermore, the red blood cells provided by transfusion could improve tissue oxygenation, while the immunomodulatory factors may regulate damaging inflammation exacerbated by low albumin levels [38]. In addition, electrolytes and nutrients bound to transfused albumin may help correct deficiencies resulting from hypoalbuminemia [39]. Finally, by increasing blood volume, transfusion may improve organ perfusion and prevent organ dysfunction that could otherwise lead to readmission in hypoalbuminemia patients [40]. However, further research is required to elucidate and confirm the exact mechanisms by which transfusion modifies the effect of low albumin on readmission. Moreover, relevant literature indicates that analyzing multiple subgroups may lead to false positive findings [41].

Given the independent association found between admission hypoalbuminemia and 30-day readmission risk after hip fracture, our institution has already incorporated serum albumin testing into perioperative assessment and risk stratification pathways. We suggest colleagues at other centers also consider integrating albumin levels into existing hip fracture protocols and risk models to enable timely identification and intervention. Moreover, the link between albumin levels and outcomes warrants evaluating serum albumin as a standardized quality metric for hip fracture programs. Further health economic studies are needed regarding potential quality improvement initiatives leveraging albumin screening to guide nutritional or medical optimization.

This study has several strengths. Firstly, the sample comprised a large cohort of elderly hip fracture patients from a level I trauma center. Comprehensive information was extracted on potential confounders to enhance adjustment. Secondly, PSM was utilized to mitigate the influence of diverse confounding factors. Thirdly, we conducted a dose–response analysis to visualize the relationship between admission hypoalbuminemia and 30-day readmission. This study enhances our understanding of admission hypoalbuminemia as an independent factor associated with 30-day readmission risk after hip fracture.

However, some limitations should be acknowledged when interpreting the results. Firstly, the retrospective single-center design has inherent biases. Secondly, information on nutritional interventions, rehabilitation, and social factors was not available. Thirdly, residual confounding cannot be fully excluded despite adjustments. Lastly, the findings require external validation in other populations. Additional prospective research is required to validate these results externally and establish causal interventions.

## Conclusions

We found that admission hypoalbuminemia was an independent predictor of increased 30-day readmission risk in elderly hip fracture patients, with a dose–response relationship between declining albumin levels and higher readmission incidence. These results highlight the potential role of serum albumin concentration as a prognostic indicator to enhance risk stratification protocols and guide management for this high-risk population.

### Supplementary Information

**Supplementary Material 1.**

## Data Availability

All the data used and analyzed during the current study are available from the corresponding author upon reasonable request.
