# Supplementary material for: Association between admission albumin levels and 30-day readmission after hip fracture surgery in geriatric patients: a propensity score-matched study
Source: BMC Musculoskelet Disord. 2024 Mar 25;25:234. doi: 10.1186/s12891-024-07336-x (PMC10962201; doi:10.1186/s12891-024-07336-x)
Supplement: Supplementary file 1 — Supplementary Material 1. [file 12891_2024_7336_MOESM1_ESM.docx]

**Supplement**

**eTable 1 Baseline characteristics of the patients by admission albumin levels(g/L) (Quartile-based four-category).**

**eTable2 Multivariate Analysis for 30-day readmission**

**eTable 1** **Baseline characteristics of the patients by admission albumin levels(g/L) (Quartile-based four-category).**

| Variables | Total  patients  (n = 1270) | admission albumin levels(g/L) | | | | P for Trend† |
| --- | --- | --- | --- | --- | --- | --- |
|  |  | Q1(< 35)  (n = 256) | Q2((35.0–37.9)  (n=300) | Q3(38.0–40.9  (n=342) | Q4(≥41)  (n = 372) |  |
| Demographic |  |  |  |  |  |  |
| Male gender (n, %) | 506 (39.8) | 111(43.4) | 114(38.0) | 127(37.1) | 154(41.4) | 0.366 |
| Age, × year (Mean, SD) | 74.69(9.55) | 79.58(8.53) | 77.85(8.27) | 73.43(9.17) | 69.94(8.29) | <0.001 |
| Smoking (n, %) | 218 (17.2) | 31(12.1) | 45(15.0) | 65(19.0) | 77(20.7) | 0.022 |
| Alcohol (n, %) | 148(11.7) | 22(8.6) | 26(8.7) | 46(13.5) | 54(14.50) | 0.030 |
| Comorbidities |  |  |  |  |  |  |
| Hypertension (n, %) | 636(50.1) | 138(53.9) | 186(62.0) | 157(45.9) | 155(41.7) | <0.001 |
| Diabetes (n, %) | 291(22.9) | 50(19.5) | 88(29.3) | 72(21.1) | 81(21.8) | 0.022 |
| COPD (n, %) | 149(11.7) | 39(15.2) | 44(14.7) | 43(12.6) | 23(6.2) | 0.001 |
| Cardiovascular disease (n, %) | 390(30.7) | 94(36.7) | 118(39.3) | 100(29.2) | 78(21.0) | <0.001 |
| Stroke (n, %) | 330(26.0) | 91(35.5) | 90(30.0) | 81(23.7) | 68(18.3) | <0.001 |
| Dementia(n, %) | 48(3.8) | 13(5.1) | 9(3.0) | 22(6.4) | 4(1.1) | 0.001 |
| Intracerebral hemorrhage (n, %) | 68(5.4) | 13(5.1) | 13(4.3) | 24(7.0) | 18(4.8) | 0.437 |
| Chronic liver disease (n, %) | 58(4.6) | 17(6.6) | 15(5.0) | 10(2.9) | 16(4.3) | 0.184 |
| Chronic kidney disease (n, %) | 64(5.0) | 16(6.3) | 22(7.3) | 15(4.4) | 11(3.0) | 0.051 |
| Operation |  |  |  |  |  |  |
| Fracture type |  |  |  |  |  |  |
| Femoral neck fracture (n, %) | 679 (53.5) | 91(35.5) | 129(43.0) | 203(59.4) | 256(68.8) | <0.001 |
| Intertrochanteric fracture (n, %) | 517(40.7) | 146(57.0) | 155(51.7) | 123(59.4) | 93(25.0) |  |
| Subtrochanteric fracture (n, %) | 74(5.8) | 19(7.4) | 16(5.3) | 16(4.7) | 23(6.2) |  |
| Surgery type |  |  |  |  |  |  |
| Total Hip Arthroplasty (n, %) | 161(12.7) | 24(9.4) | 23(7.7) | 48(14.0) | 66(17.7) | <0.001 |
| Hemiarthroplasty (n, %) | 319(25.1) | 52(20.3) | 83(27.7) | 99(28.9) | 85(22.8) |  |
| Intramedullary nail fixation (n, %) | 414(32.6) | 113(44.1) | 128(42.7) | 93(27.2) | 80(21.5) |  |
| Internal fixation with steel plate (n, %) | 168(13.2) | 48(18.8) | 42(14.0) | 42(12.3) | 36(9.7) |  |
| Internal fixation with hollow nails (n, %) | 208(16.4) | 19(7.4) | 24(8.0) | 60(17.5) | 105(28.2) |  |
| Intraoperative blood loss, ×ml (Mean, SD) | 175.30(152.90) | 196.56(158.95) | 182.83(127.91) | 170.04(171.39) | 159.45(147.58) | 0.018 |
| Transfusion (n, %) | 210(16.5) | 64(25.0) | 70(23.3) | 41(12.0) | 35(9.4) | <0.001 |
| Postoperative ICU (n, %) | 63(5.0) | 26(10.2) | 17(5.7) | 13(3.8) | 7(1.9) | <0.001 |
| Admission time |  |  |  |  |  |  |
| ＜6 hours (n, %) | 672(52.9) | 115(44.9) | 162(54.0) | 182(53.2) | 213(57.3) | <0.001 |
| 6-24 hours (n, %) | 195(15.4) | 32(12.5) | 54(18.0) | 68(19.9) | 41(11.0) |  |
| ≥ 24 hours (n, %) | 403(31.7) | 109(42.6) | 84(28.0) | 92(26.9) | 118(31.7) |  |
| Bedridden time, ×day (Mean, SD) | 5.90(4.03) | 6.77(5.04) | 6.43(4.23) | 5.63(3.44) | 5.12(3.38) | <0.001 |
| Intraoperative time, ×hour (Mean, SD) | 1.67(0.80) | 1.77(0.86) | 1.60(0.70) | 1.62(0.75) | 1.69(0.88) | 0.059 |
| ASA classification |  |  |  |  |  |  |
| Ⅲ-Ⅳ (n, %) | 710(55.9) | 180(70.3) | 197(65.7)) | 177(51.8) | 156(41.9) | <0.001 |
| Ⅰ-Ⅱ (n, %) | 560(44.1) | 76(29.7) | 103(34.3) | 165(48.2) | 216(58.1) |  |
| Laboratory findings |  |  |  |  |  |  |
| WBC count, ×10^9/L (Mean, SD) | 8.84(2.86) | 8.79(3.24) | 8.88 (3.04) | 8.75(2.52) | 8.92(2.72) | 0.842 |
| HGB level, ×g/L (Mean, SD) | 119.80(20.63) | 106.38(20.91) | 115.30(17.98) | 121.22(18.17) | 131.35(17.72) | <0.001 |
| Blood glucose, ×mmol/L (Mean, SD) | 6.92(2.65) | 6.81(2.36) | 7.36(2.98) | 6.83(2.63) | 6.74(2.54) | 0.014 |
| BUN,×mmol/L (Mean, SD) | 7.39(4.85) | 8.12(4.15) | 8.45(7.53) | 6.89(3.28) | 6.51(3.27) | <0.001 |
| Cr, ×umol/L (Mean, SD) | 72.35(64.38) | 74.23(48.61) | 82.16(96.38) | 70.19(68.51) | 65.12(24.84) | 0.006 |
| D-Dimer, ×mg/L (Mean, SD) | 8.84(2.86) | 4.85(4.60) | 5.13(4.91) | 5.01(5.06) | 4.59(5.40) | 0.530 |
| Common complication |  |  |  |  |  |  |
| DVT (n, %) | 164(12.9) | 41(16.0) | 40(13.3) | 40(11.7) | 43(11.6) | <0.001 |
| UTI (n, %) | 294(23.1) | 88(34.4) | 85(28.3) | 70(20.5) | 51(13.7) | <0.001 |
| Pneumonia (n, %) | 115(9.1) | 36(14.1) | 35(11.7) | 26(7.6) | 18(4.8) | <0.001 |

† P values for linear trend for continuous variables are from a generalized linear model, and categorical variables are from an ordinal or logistic regression

Abbreviations: COPD, Chronic obstructive pulmonary disease; ASA: the American Society of Anesthesiologists Physical Status Classification System; WBC, White blood cell; HGB, hemoglobin; RBC, red blood cell; WBC, White blood cell; BUN, Blood urea nitrogen ; Cr, Creatinine

**eTable2 Multivariate Analysis for 30-day readmission**

| Variables | Univariate | | | Multivariate | | |
| --- | --- | --- | --- | --- | --- | --- |
|  | OR | 95%CI | p-value | OR | 95%CI | p-value |
| Demographic |  |  |  |  |  |  |
| Male gender (n, %) | 0.83 | 0.60-1.14 | 0.250 | <NA> | <NA> | <NA> |
| Age, × year (Mean, SD) | 1.07 | 1.05-1.09 | <0.001 | 1.05 | 1.03-1.07 | <0.001 |
| Smoking (n, %) | 0.79 | 0.51-1.23 | 0.301 | <NA> | <NA> | <NA> |
| Alcohol (n, %) | 0.79 | 0.47-1.32 | 0.364 | <NA> | <NA> | <NA> |
| Comorbidities |  |  |  |  |  |  |
| Hypertension (n, %) | 1.85 | 1.34-2.54 | <0.001 | 1.21 | 0.85-1.72 | 0.239 |
| Diabetes (n, %) | 1.81 | 1.29-2.54 | 0.001 | 1.35 | 0.87-2.09 | 0.239 |
| COPD (n, %) | 1.56 | 1.01-2.41 | 0.045 | 0.91 | 0.56-1.49 | 0.785 |
| Cardiovascular disease (n, %) | 1.48 | 1.07-2.05 | 0.017 | 0.83 | 0.58-1.19 | 0.267 |
| Stroke (n, %) | 2.38 | 1.73-3.30 | <0.001 | 1.57 | 1.10-2.23 | 0.011 |
| Dementia(n, %) | 1.36 | 0.65-2.86 | 0.414 | <NA> | <NA> | <NA> |
| Intracerebral hemorrhage (n, %) | 2.04 | 1.15-3.61 | 0.015 | 2.08 | 1.12-3.85 | 0.020 |
| Chronic liver disease (n, %) | 1.74 | 0.92-3.28 | 0.090 | <NA> | <NA> | <NA> |
| Chronic kidney disease (n, %) | 2.03 | 1.13-3.66 | 0.018 | 1.74 | 0.91-3.25 | 0.096 |
| Operation |  |  |  |  |  |  |
| Fracture type | 1.14 | 0.88-1.48 | 0.332 | <NA> | <NA> | <NA> |
| Surgery type | 0.81 | 0.71-0.92 | 0.001 | 0.87 | 0.74-1.02 | 0.080 |
| Intraoperative blood loss, | 1.00 | 1.00-1.00 | 0.453 | <NA> | <NA> | <NA> |
| Transfusion | 1.20 | 0.81-1.80 | 0.365 | <NA> | <NA> | <NA> |
| Postoperative ICU | 1.11 | 0.55-2.21 | 0.778 | <NA> | <NA> | <NA> |
| Admission time | 1.12 | 0.94-1.33 | 0.202 | <NA> | <NA> | <NA> |
| Bedridden time | 1.02 | 0.98-1.06 | 0.301 | <NA> | <NA> | <NA> |
| Intraoperative time | 0.83 | 0.67-1.03 | 0.096 | <NA> | <NA> | <NA> |
| ASA classification | 3.14 | 2.18-4.52 | <0.001 | 1.71 | 1.14-2.55 | 0.009 |
| Laboratory findings |  |  |  |  |  |  |
| WBC count | 1.05 | 0.99-1.10 | 0.099 | <NA> | <NA> | <NA> |
| HGB level | 0.99 | 0.98-0.99 | <0.001 | 1.00 | 0.99-1.01 | 0.785 |
| Blood glucose | 1.12 | 1.07-1.18 | <0.001 | 1.07 | 1.01-1.15 | 0.040 |
| BUN | 1.03 | 1.00-1.06 | 0.036 | 0.99 | 0.95-1.02 | 0.449 |
| Cr | 1.00 | 1.00-1.00 | 0.081 | <NA> | <NA> | <NA> |
| D-Dimer | 1.02 | 0.99-1.05 | 0.241 | <NA> | <NA> | <NA> |
| Albumin | 0.90 | 0.87-0.93 | <0.001 | 0.94 | 0.90-0.98 | 0.003 |
| Common complication |  |  |  |  |  |  |
| DVT (n, %) | 1.64 | 1.08-2.49 | 0.019 | 1.08 | 0.67-1.73 | 0.755 |
| UTI (n, %) | 1.53 | 1.09-2.16 | 0.015 | 0.82 | 0.55-1.22 | 0.323 |
| Pneumonia (n, %) | 3.05 | 1.99-4.69 | <0.001 | 1.83 | 1.12-2.99 | 0.016 |

p-value is from Univariate and Multivariate (P<0.05 indicates statistical significance)

Abbreviations: COPD, Chronic obstructive pulmonary disease; ASA: the American Society of Anesthesiologists Physical Status Classification System; WBC, White blood cell; HGB, hemoglobin; RBC, red blood cell; WBC, White blood cell; BUN, Blood urea nitrogen ; Cr, Creatinine
